# Avasimibe Alleviates Disruption of the Airway Epithelial Barrier by Suppressing the Wnt/β-Catenin Signaling Pathway

**DOI:** 10.3389/fphar.2022.795934

**Published:** 2022-02-11

**Authors:** Zicong Zhou, Shixiu Liang, Zili Zhou, Jieyi Liu, Xiaojing Meng, Fei Zou, Changhui Yu, Shaoxi Cai

**Affiliations:** ^1^ Chronic Airways Diseases Laboratory, Department of Respiratory and Critical Care Medicine, Nanfang Hospital, Southern Medical University, Guangzhou, China; ^2^ Department of Occupational Health and Occupational Medicine School of Public Health, Southern Medical University, Guangzhou, China

**Keywords:** allergic asthma, avasimibe, basal cell, epithelial barrier, Wnt/β-catenin

## Abstract

Avasimibe (Ava) is an acetyl-CoA acetyltransferase 1 (ACAT1) specific inhibitor and an established medicine for atherosclerosis, owing to its excellent and safe anti-inflammation effects in humans. However, its efficacy in asthma has not yet been reported. We first administered varying concentrations of avasimibe to house dust mite (HDM)-induced asthmatic mice; results showed that 20 mg/kg avasimibe most significantly reduced IL-4 and IL-5 production in bronchoalveolar lavage fluid (BALF) and total IgE in serum, and the avasimibe treatment also exhibited lower mucus secretion, decreased goblet and basal cells but increased ciliated cells compared to the HDM group. And the redistribution of adherens junction (AJ) proteins induced by HDM was far more less upon avasimibe administration. However, avasimibe did not reduce the cholesterol ester ratio in lung tissues or intracellular cholesterol ester, which is avasimibe’s main effect. Further analysis confirmed that avasimibe impaired epithelial basal cell proliferation independent of regulating cholesterol metabolism and we analyzed datasets using the Gene Expression Omnibus (GEO) database and then found that the KRT5 gene (basal cell marker) expression is correlated with the β-catenin gene. Moreover, we found that β-catenin localized in cytomembrane upon avasimibe treatment. Avasimibe also reduced β-catenin phosphorylation in the cytoplasm and inactivated the Wnt/β-catenin signaling pathway induced by HDMs, thereby alleviating the airway epithelial barrier disruption. Taken together, these findings indicated that avasimibe has potential as a new therapeutic option for allergic asthma.

## Introduction

Allergic asthma is a common, complex chronic pulmonary disease, characterized by bronchoconstriction, reversible airways hyperresponsiveness, and sensitivity to inhaled environmental irritants (Finotto et al., 2001; Percopo et al., 2014; Drake et al., 2018). However, current therapeutic strategies for asthma mostly only give a symptomatic relief (Duvall et al., 2017). To address the underlying pathogenesis, many researchers have engaged in studies that focused on inhibitors of critical factors, such as anti-IL-4, anti-IL-33, or targeting TSLP/TSLPR axis, to be therapeutically beneficial in asthma (Qin et al., 2015; Emdin et al., 2018). Although they have obtained numerous results, there is still a long distance from the laboratories to the clinical treatments.

Many studies have shown that environmental agents’ chronic repetitive insult asthmatic subjects causing disruption of the airway epithelial barrier, which serves as the first line of defense against environmental stresses, owing to the fact that they cause structural dysfunction of the adherens junctions (AJs) and tight junctions (TJs), basal cells proliferating and differentiating into goblet cells with mucus hypersecretion, thereby disrupting the epithelial barrier. Notably, desquamation and denudation of the epithelium are significant features of the airway epithelial barrier disruption in asthma (Finotto et al., 2001; Lundblad et al., 2011; Drake et al., 2018; Wei et al., 2020).

In recent years, numerous research efforts have been directed to unraveling the relationship between lipid metabolism and asthma. Notably, recent studies have shown that the homeostasis of cellular cholesterol, a critical component of lipids and an essential lipid component in mammalian cell plasma membranes, affects inflammation in the airway (Lagace, 2015). Consequently, statins, the cholesterol-lowering medicines, have been used to reduce intracellular cholesterol synthesis and alleviate the associated airway inflammation (He et al., 2020). Total cholesterol comprises free cholesterol (FC) and cholesterol ester (CE), with cholesterol ester acting as an intracellular storage form. Disrupting cholesterol homeostasis that accumulates free cholesterol in cells may lead to plasma membrane breakdown, apoptosis, and even augmentation of inflammatory response in an organism (Erbay et al., 2009; Song et al., 2016; Nolan et al., 2018). To date, only a handful of studies have described the interaction between cholesterol homeostasis and allergic asthma.

Avasimibe (Ava), a drug that specifically targets acetyl-CoA acetyltransferase 1 (ACAT1), has been shown to reduce cholesterol ester transformation. Currently, the drug is an established medicine for clinical treatment of coronary atherosclerosis, owing to its anti-inflammatory efficacy and excellent safety profile. On the other hand, avasimibe exhibits potential antitumor effects that impair proliferation and viability of tumor cells (Gao et al., 2021), and enhance the antitumor activity of mouse CD8^+^ effector T cells, therefore suppressing tumor growth (Börtlein et al., 2019). In recent years, accumulating evidence showed that avasimibe functions in various diseases through its anti-inflammatory and antitumor growth effects, but the correlation between the drug and allergic asthma has not been reported (Wegiel et al., 2018; Jiang et al., 2019; Liu et al., 2020).

Chronic airway inflammation is one of the most important features in asthma; we wondered whether avasimibe exerts anti-inflammatory function as well in allergic asthma, and thereby provides a new therapeutic option in clinical application.

## Materials and Methods

### Animal and Experimental Protocol

Specific pathogen-free (SPF) C57BL/6 mice (female, 4–6 weeks old) were purchased from the Center of Experimental Animals of Southern Medical University (Guangzhou, China). The mice were kept in the SPF environment, at 24°C, 40–70% humidity, and half day light/dark cycle and fed using a standard sterilized mouse feed with water provided ad libitum. House dust mite (HDM) extract was purchased from Greer Laboratories (United States). All experiments were conducted in accordance with the guidelines outlined by the Committee of Southern Medical University on the Use and Care of Laboratory Animals. The mice were randomly assigned to the following five groups: 1) the control, injected with sterilized phosphate-buffered saline (PBS) (Gibco, United States); 2) the HDM group, intratracheally administered with HDMs (25 µg/mouse, dissolved in sterilized PBS); (3)10 mg/kg avasimibe (Selleck, United States), intraperitoneally injected 1 h before HDM administration; 4) 20 mg/kg avasimibe, intraperitoneally injected 1 h before HDM administration; and 5) 30 mg/kg avasimibe, intraperitoneally injected 1 h before HDM administration. The HDM-induced asthmatic mouse model was established as follows: mice were first sensitized by intraperitoneal injection with 25 µg HDMs, dissolved in sterilized PBS, once a week for total 2 weeks. Seven days after the last sensitization, the mice were treated with 25 µg HDMs, three times in 3 days. Finally, the mice were killed 24 h after the last challenge, and samples were harvested.

### Measurement of Airway Hyperresponsiveness

Mice were anesthetized by intraperitoneal injection of 2,2,2-tribromoethanol (Sigma Aldrich, United States) and their tracheas cannulated, followed by the measurement of baseline airway resistance (performed using a DSITM Buxco^®^ PFT Controller, United States). Next, aerosol inhaled increasing doses (3.125, 6.25, 12.5, 25, and 50 mg/ml) of methacholine (Sigma Aldrich, United States) before each measurement. The airway resistance values were marked down from the measuring machine, and a line graph was mapped by GraphPad Prism 7.

### Cell Cultures and Treatment

The human bronchial epithelial cell line, HBE-135° (ATCC, United States), was inoculated in a Keratinocyte Medium (ScienCell, United States) and incubated using a humidified incubator at 37 °C with an atmosphere of 5% CO2. Upon reaching 70–80% density, the cells were digested with trypsin and then seeded into 6-well culture plates at an appropriate density for experiments. Upon reaching a confluence of 90%, the cells were rinsed twice with PBS and then stimulated with 20 µg HDMs, or administered with avasimibe (Selleck, United States) 1 h before HDM administration.

### Determination of Concentrations of Inflammatory Cells and Cytokines in Bronchoalveolar Lavage Fluid

The bronchoalveolar lavage was performed in anesthetized mice, by slowly injecting them with 800 µl of PBS through a tracheotomy tube and then gently withdrawing the PBS. This activity was repeated twice. Next, 10 µl of BALF was collected and used to count total cells using a TC 20™ Automated Cell Counter (BIO-RAD, United States). The remaining sample was centrifuged for 10 min at 4°C, 3,000 rpm and the supernatant immediately stored at -80°C before the determination of cytokine concentration. The pellet was resuspended in 100 µl of 4% neutral formalin, and 10 µl was taken for smearing. To assess inflammatory cells, the smears were stained with hematoxylin−eosin (HE) and eosinophil cells ratio determined. Cytokine concentration, for IL-4 and IL-5, was also performed on the supernatant using LUMINEX multi-factor detection (MERCK, United States).

### Measurement of Serum IgE

Blood was drawn from killed mice and centrifuged at 3,000 rpm for 10 min at 4°C, to obtain serum. Next, serum IgE was measured via LUMINEX multi-factor detection (MERCK, United States).

### Primary Mouse Airway Epithelial Basal Cells Harvest and Culture

The SPF C57BL/6 mice (4 weeks old) were killed by subjecting them to an overdose of inhaled 4–5% isoflurane. The mice were surface sterilized using 75% alcohol for at least 10 min and then placed on a sterilized desk. Next, a 1.5-cm longitudinal skin incision was made along the midline of the neck to expose the trachea, and then the trachea was cut off from the thyroid to the hilus pulmonis. The separated trachea was washed in precold sterilized PBS buffer to remove mucus and blood and then transferred to a harvesting medium (F-12 without FBS, Gibco, United States). The tissues were cut into small pieces (∼1 mm) using scissors and then transferred to a digestion medium (harvest medium with protease from *Streptomyces* griseus, Sigma-Aldrich, United States). The ixture was shaken and then left to stand for 18 h at 4°C. Digestion was terminated by the addition of a harvesting medium with 10% FBS, mixed by turning the tube upside down 30 times. The mixed medium without tissues was collected, and the activity was repeated three times. The contents were centrifuged at 600 xg for 5 min, pellets collected, and resuspended in 2 ml DMEM (Gibco, United States) supplemented with 10% FBS. The cells were seeded into 6-cm petri dishes and cultured for 2 h using a humidified incubator at 37°C with an atmosphere of 5% CO2. After incubation, the medium with non-adherent cells was collected and centrifuged for 5 min at 600 xg, and pellets were collected and then resuspended in 2 ml proliferation medium. The resultant cells were seeded in a 3.5-cm petri dishes precoated with collagen (Sigma-Aldrich, United States) for subsequent experiments.

### Histological Examination of Pulmonary Tissues

Left lung tissues were fixed in 4% neutral formalin for at least 48 h, embedded in paraffin and then cut into 5-µm sections using a Leica microtome 2030 (Leica Microsystems Nussloch GmbH, Germany). The sections were deparaffinized, stained with hematoxylin−eosin, and observed under a microscope. The morphology was evaluated using the following criterion: 0, no infiltration of inflammatory cells into the airway submucosa; 1, one round of inflammatory cells infiltration; 2, two rounds of inflammatory cells infiltration; 3, three rounds of inflammatory cells infiltration; and 4, four or more rounds of inflammatory cells infiltration. Next, we used periodic acid–schiff (PAS) to detect mucus production based on the following equation: ((area of PAS staining) x (mean intensity of PAS staining))/total area of airway epithelia. For immunohistochemistry, standard deparaffinized sections were submerged in pre-boiled citrate buffer (pH = 6.0) for antigen retrieval, blocked with 3% H2O2 for 10 min at room temperature (to block endogenous peroxidase activity), followed by overnight incubation with 1:100 dilutions of anti-E-cadherin (Cell Signaling Technology, United States), anti-β-catenin (Cell Signaling Technology, United States), and 1:200 dilutions of anti-α-SMA (Abcam, United States) at 4°C. The contents were washed three times with PBS, slices incubated with the secondary antibody for 30 min at room temperature, and signals detected using a DAB peroxidase substrate kit (ZhongShanJinQiao, China).

### Western Blot Analysis

Treated cells were lysed for 20 min using the RIPA buffer (KeyGEN Biotech, China) containing PMSF (KeyGEN Biotech, China) at 4°C. Lung tissues were lysed in RIPA buffer (KeyGEN Biotech, China) containing PMSF (KeyGEN Biotech, China) at 4°C and sonicated for another 6 × 30 s at high intensity. Lysed samples were centrifuged at 12,000 rpm for 10 min at 4°C, supernatants were collected and boiled with standard 5x SDS sample buffer. Plasma membrane protein extraction was done using the Minute™ Plasma Membrane Protein isolation and Cell Fractionation Kit (Invent, United States), according to the manufacturer’s instructions. The samples were resolved via SDS-PAGE, transferred to PVDF membranes before incubation with the following primary antibodies: anti-MUC5AC (Abcam, United States), anti-FOXJ1 (Invitrogen, United States), anti-KRT5 (Abcam, United States), anti-caspase-3 (Cell Signaling Technology, United States), cleaved caspase-3 (Cell Signaling Technology, United States), anti-E-cadherin (Cell Signaling Technology, United States), anti-ATP1A1 (Proteintech, China), anti-β-actin (Proteintech, China), anti-β-tublin (Proteintech, China), anti-lamin B1 (HuaBio, China), and β-Catenin Antibody Sampler Kit (Cell Signaling Technology, United States). The membranes were then incubated with the secondary antibody (IRDye 800CW or IRDye 680RD, United States), and signal intensities were analyzed using an Odyssey^®^ Imaging System (Licor, United States).

### Quantitative Real-Time Polymerase Chain Reaction

Total RNA was extracted from treated cells using the Cell Total RNA Isolation Kit (Foregene, China), according to the manufacturer’s instructions. The RNA was reverse-transcribed to first-strand complementary DNA (cDNA) using the PrimeScriptTM RT reagent kit (Takara, Japan) according to manufacturer’s instructions. The cDNA was subjected to qRT-PCR using the SYBR Green kit (Yeasen, China) targeting genes, as outlined in Table 1, performed using a real-time PCR instrument (Bio-Rad, United States). Levels of mRNA expression were normalized to those of GAPDH (internal control), calculated using the ΔCt method and calculated as 2−ΔCt values.

**TABLE 1:** Primer sequences for genes used for qRT-PCR.

Name		5′→3′	aa
Human			
MUC5AC	F	TGCCCCTACAACAAGAACAAC	21
	R	GGAACAGCACTGGGAGTAGTT	21
FOXJ1	F	GCCTCCCTACTCGTATGCCA	20
	R	GCCGACAGGGTGATCTTGG	19
KRT5	F	AGGAGTTGGACCAGTCAACAT	21
	R	TGGAGTAGTAGCTTCCACTGC	21
AQP3	F	GGGGAGATGCTCCACATCC	19
	R	AAAGGCCAGGTTGATGGTGAG	21
SLC12A2	F	TAAAGGAGTCGTGAAGTTTGGC	22
	R	CTTGACCCACAATCCATGACA	21
SCNN1	F	AGGGGAACAAGCGTGAGGA	19
	R	GGTGGAACTCGATCAGGGC	19
GAPDH	F	GGAGCGAGATCCCTCCAAAAT	21
	R	GGCTGTTGTCATACTTCTCATGG	23
Mouse			
FOXJ1	F	CCCTGACGACGTGGACTATG	20
	R	GCCGACAGAGTGATCTTGGT	20
MUC5AC	F	GTGGTTTGACACTGACTTCCC	21
	R	CTCCTCTCGGTGACAGAGTCT	21
KRT5	F	TCTGCCATCACCCCATCTGT	20
	R	CCTCCGCCAGAACTGTAGGA	20
GAPDH	F	GGCAAATTCAACGGCACAGTCAAG	24
	R	TCGCTCCTGGAAGATGGTGATGG	23

### Immunofluorescence

Cells were seeded onto glass-bottomed plates, at appropriate densities, and then incubated using a humidified incubator at 37°C with an atmosphere of 5% CO2. Upon reaching a 90% confluence, the cells were treated with the aforementioned reagents and then fixed with 4% paraformaldehyde at room temperature for 30 min. Thereafter, the cells were rinsed three times with PBS for 5 min, blocked for 30 min with 5% BSA in PBS at room temperature, and the cell monolayers were incubated overnight with the primary antibody at 4°C in PBS containing 5% BSA, primary rabbit anti-E-cadherin (Cell Signaling Technology, United States), and anti-β-catenin (Santa Cruz, United States) antibodies. The cells were washed three times with PBS, incubated for 1 h with the secondary antibody, Alexa Fluor 488 (R37118) or Alexa Fluor 594 (R37119) (1:200 diluted in PBS containing 5% BSA) (Invitrogen, United States), at room temperature in darkness. Cell nuclei were stained for 2 min with DAPI (Sigma Aldrich, United States) at room temperature, and images were captured using a confocal microscope (FV1000, Olympus, Japan).

### Evaluation of Epithelial Barrier Function

Epithelial barrier integrity was assessed by measuring transepithelial electrical resistance (TEER) and FITC-Dx flux across the monolayers of cultured epithelial cells. Briefly, confluent monolayers of HBECs, polarized at an air–liquid interface (ALI), and were cultured in 24-well transwell inserts (Corning Costar, United States). TEER was measured using a Millicell ERS-2 Epithelial Volt-Ohm meter with an STX01 electrode (Millipore Corp, Billerica, MA, United States). Next, the apical medium (luminal side) was replaced with 200 µl of phenol red-free RPMI 1640 containing 0.5 mg/ml FITC-Dx (Sigma, United States), and the basal medium (non-luminal side) was replaced with 500 µl of phenol red-free RPMI 1640 without FITC-Dx, with a 90-min incubation at 37°C. Samples were analyzed by fluorimetry, at an excitation of 492 nm and an emission of 530 nm. Finally, epithelial permeability was expressed as the percent leakage of FITC-Dx from apical to basolateral compartments.

### Flow Cytometry

Apoptosis was analyzed by incubating the cells using an Annexin V-EGFP Apoptosis Detection Kit (KeyGen, China) according to manufacturer’s instructions, and then examined using a flow cytometer.

### Cholesterol Assay

Cholesterol concentration was measured using the Free Cholesterol Assay and Total Cholesterol Assay (Applygen Technologies, China) Kits, according to manufacturer’s instructions.

### Cell Viability and Proliferation Assay

EdU proliferation assay was carried out using the BeyoClick™ EdU Cell Proliferation Kit (Beyotime, China) according to manufacturer’s instructions, While cell viability, and proliferation assay was performed using the Cell Counting Kit-8 (CCK8, Dojindo, Japan) according to manufacturer’s instructions.

### TUNEL Staining

The TUNEL staining was carried out using a One-Step TUNEL Apoptosis Assay Kit (KeyGen, China), according to the manufacturer’s instructions.

### GEO Analysis

Datasets were downloaded from the GEO database (https://www.ncbi.nlm.nih.gov/geo/), in an MINIML format. Box plots and multi-gene correlation graphs were generated using the “ggplot2” and “pheatmap” packages implemented in R software.

### Statistical Analysis

Statistical analysis was performed using GraphPad Prism 7 software, and data were expressed as means ± standard deviations (SD). Differences among groups were analyzed using one-way analysis of variance (ANOVA) accompanied by Bonferonni’s post hoc test f. Data followed by *p* < 0.05 were considered statistically significant.

## Results

### Avasimibe Improves Airway Inflammation in HDM-Induced Allergic Asthma *in vivo*

**FIGURE 1:**
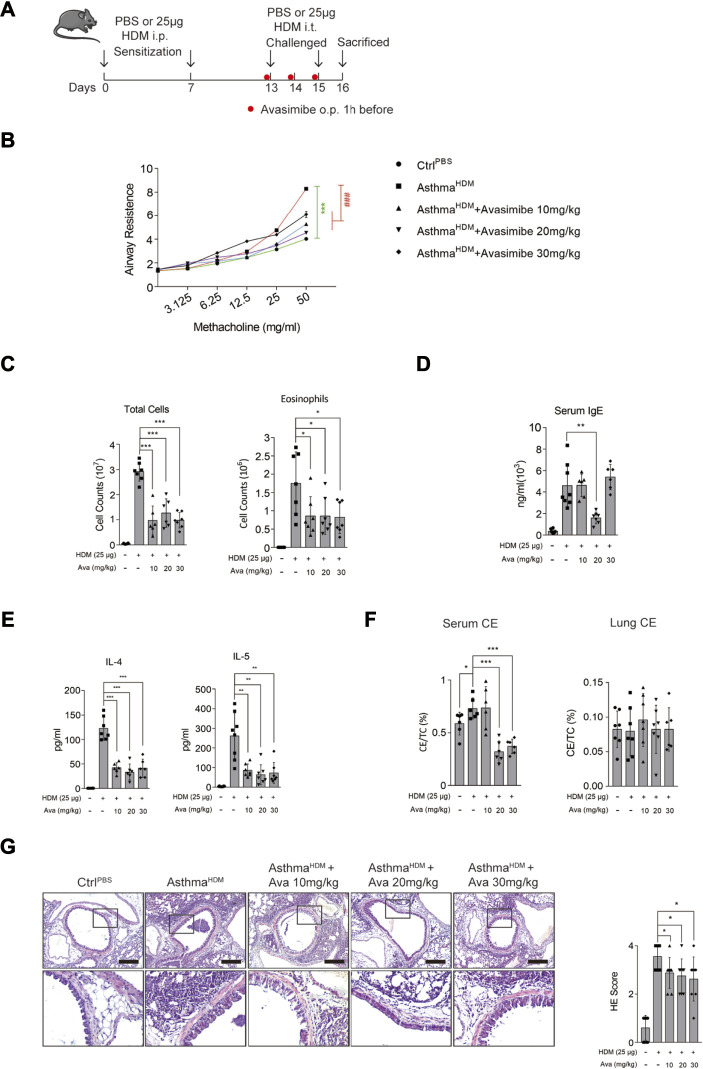
Avasimibe suppresses inflammation in HDM-induced allergic asthma *in vivo*. **(A)** (Left panel) Establishment of the HDM-induced allergic asthma mouse model. (Right panel) Different shape dots represent different groups. **(B)** Changes in airway resistance (Rl) in response to increasing dose of methacholine (MCh). **(C)** Bronchoalveolar lavage fluid (BALF) total cells and eosinophilic cells count. **(D)** Levels of total serum IgE. **(E)** IL-4 and IL-5 concentrations in Bronchoalveolar lavage fluid (BALF). **(F)** The cholesterol ester (CE) ratio in serum or lung tissues. **(G)** (Left panel) HE stained sections in lung tissues (scale bar = 200 µm). (Right panel) HE scores. Data presented are means ± SD. n ≥ 6, *p < 0.05, **p < 0.001 by one-way ANOVA.

We used an HDM sensitization/challenge to establish an asthmatic mouse model (Figure 1A). Mice were anesthetized, 24 h after the last challenge, and their airway resistance was measured. Results showed that HDM-induced mice that had inhaled methacholine had significantly higher airway resistance than those in the control and three Ava-treating groups (Figure 1B). Next, mice were euthanized after measurement of airway resistance, and samples were harvested. To assess airway inflammation, we collected and analyzed the bronchoalveolar lavage fluid (BALF) and lung tissues. Results showed that mice in the HDM-induced group had a significantly higher number of total cells and eosinophils than those in the control and three Ava-treating groups (Figure 1C). We further isolated serum from the blood samples and found that a significantly higher concentration of serum IgE in the HDM-induced than the control group. However, the serum IgE from mice in the Ava-treating group (20 mg/kg) was significantly lower than that in the HDM-induced group (Figure 1D). Notably, IL-4 and IL-5 in BALF of HDM-induced mice were significantly higher than that in the control group but markedly lower in three Ava-treating groups (Figure 1E). Next, we assessed infiltration of airway inflammatory cells using HE staining and found that the infiltration in the HDM-induced group was significantly higher compared to control group, with Ava-treatment alleviating this phenomenon (Figure 1G). We also observed elevated HDM-induced mucus secretion, which was improved by avasimibe administration (20 and 30 mg/kg) (Supplementary Figure S1A). Overall, these results indicated that 20 mg/kg avasimibe significantly attenuated airway inflammation in allergic asthma.

### Avaismibe Alleviates HDM-Induced Airway Epithelial Barrier Disruption *In Vivo*

**FIGURE 2:**
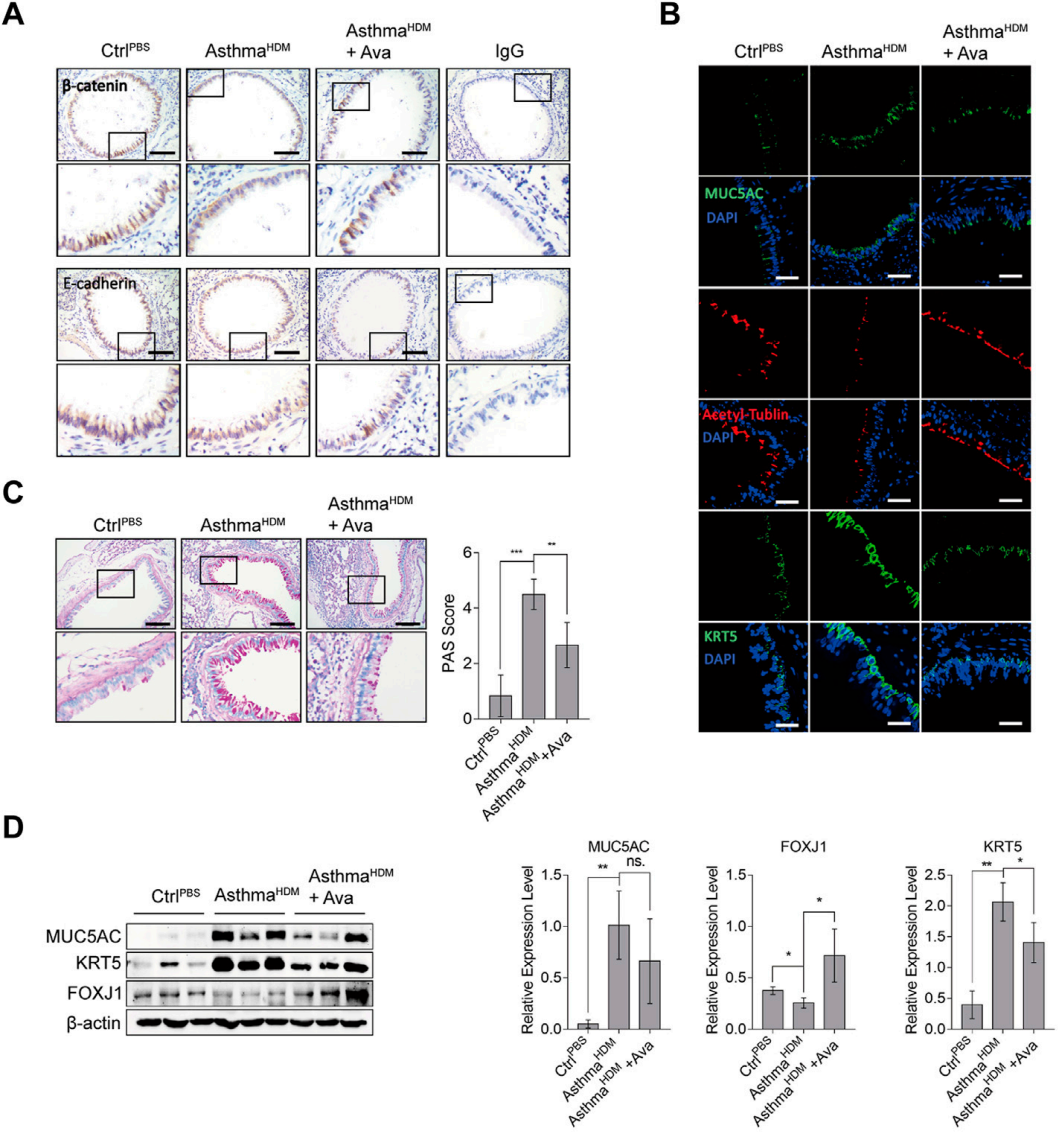
Avasimibe improves disruption of the airway epithelial barrier. **(A)** Immunohistochemical staining distribution of β-catenin and E-cadherin. Scale bar = 200 µm. **(B)** Immunofluorescence staining of frozen lung sections for MUC5AC (goblet cell), acetyl-tublin (ciliated cell), and KRT5 (basal cell). Scale bar = 200 µm. **(C)** Periodic acid–schiff (PAS) staining for mucus in airway epithelia. Evaluation was according to the criterion mentioned in Methods. **(D)** Western blots showing levels of MUC5AC, KRT5, and FOXJ1 proteins in lung tissues in three groups. Data presented are means ± SD. n ≥ 3, *p < 0.05, **p < 0.001 by one-way ANOVA.

Our previous research has confirmed that HDM induces structural disruption of adherens junction in epithelia (Dong et al., 2016; Zhang et al., 2017; Dong et al., 2017; Ye et al., 2019). In the present study, treatment with 20 mg/kg avasimibe caused an excellent anti-inflammatory effect, and there was no significant toxicity on lung tissues upon the distinct concentration, since the apoptotic cells in the lung decreased in the Ava-treated group compared to the HDM-induced group (Supplementary Figure S4). Thus, we selected this concentration for further analysis. Subsequently, we performed immunohistochemistry and immunofluorescence analyses to assess expression of epithelial markers and estimate airway epithelial barrier dysfunction. Results revealed a significantly higher redistribution of β-catenin and E-cadherin in the HDM-induced group relative to the control group, while the Ava-treated group showed markedly improved HDM-induced β-catenin and E-cadherin structural disruption in airway epithelia (Figure 2A). The airway epithelial cells mainly consist of ciliated cells, mucin cells, basal cells, and other functional cells with different markers, respectively (Vieira Braga et al., 2019; Yoshie et al., 2019; Zaragosi et al., 2020). Therefore, we used those markers, MUC5AC for goblet cells, FOXJ1 and acetyl-tublin for ciliated cells, and KRT5 for basal cells, and we found significantly higher proliferation of goblet and basal cells but significantly lower ciliated cells in the HDM-induced group than the control group. In addition, treatment with avasimibe exhibited significantly improved HDM-induced epithelia disruption and remodeling process (Figure 2B). The PAS staining revealed production of mucus in mice in the airway in the HDM-induced group compared with the control group, although the phenomenon was significantly lower in mice in the Ava-treating group (Figure 2C). Western blots, targeting expression of MUC5AC, KRT5, and FOXJ1 in lung tissues, revealed significant downregulation of MUC5AC and KRT5 but the upregulation of FOXJ1 in Ava-treated relative to the HDM-induced group (Figure 2D). Results of mRNA expression, targeting MUC5AC, KRT5, and FOXJ1, were consistent with the findings of protein expression, indicating that avasimibe administration impaired the differentiation capability of basal cells (Supplementary Figure S1C). In addition, the immunohistochemistry was used to detect α-SMA, for airway smooth cells, in the airway submucosa, and we surprisingly found that 20 mg/kg Ava administration significantly reduced HDM-induced hyperplasia of smooth cells (Supplementary Figure S5). Overall, these results suggested that HDMs induced airway disruption, while avasimibe treatment could improve this phenomenon.

### Avasimibe Does Not Regulate Cholesterol Metabolism in Lung Tissues and Airway Epithelial Cells

**FIGURE 3:**
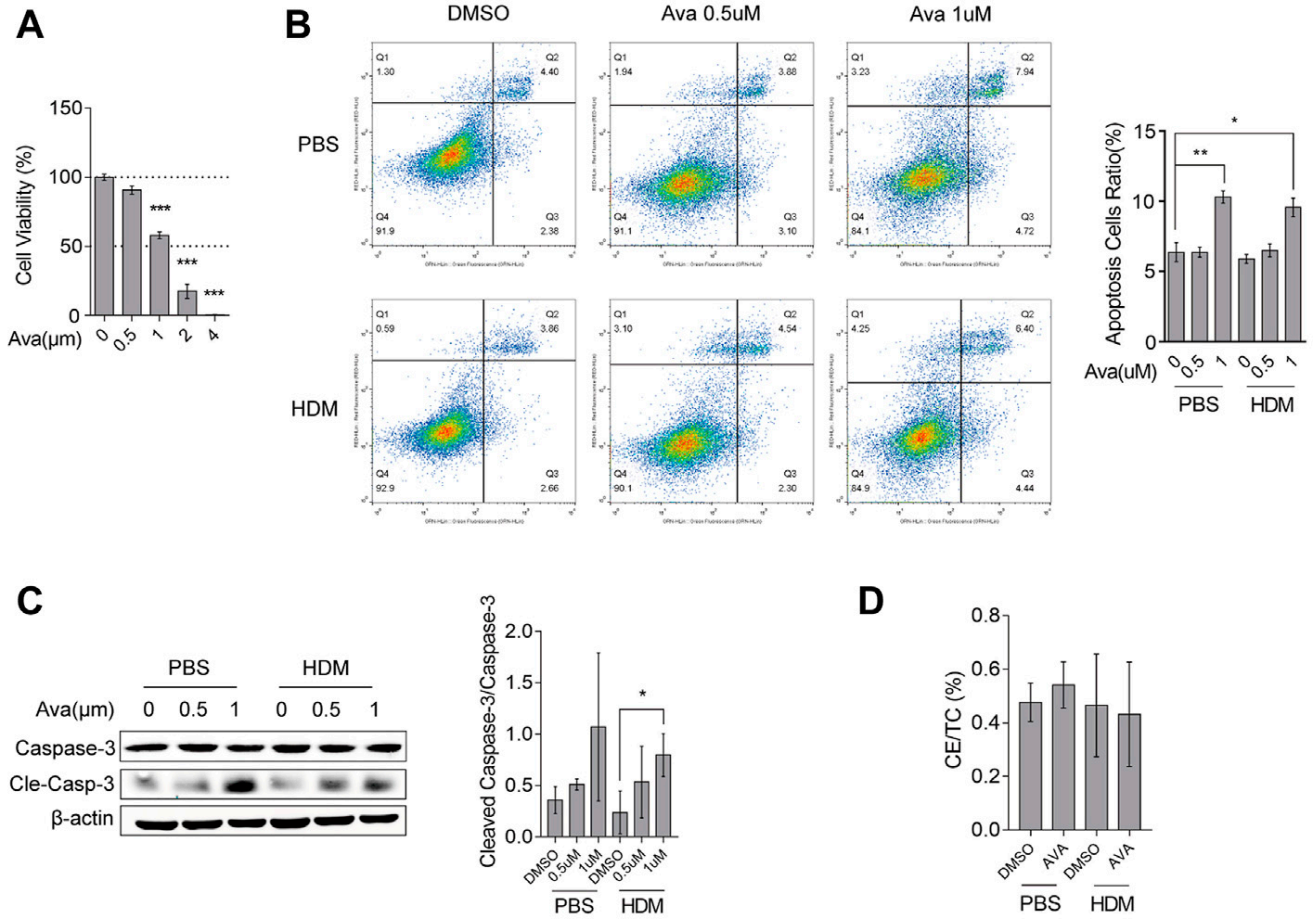
Avasimibe does not affect cholesterol metabolism in HBECs. **(A)** Viability of HBECs assessed using the CCK8 assay. **(B)** Levels of cell apoptosis after flow cytometric analysis. **(C)** Western blots showing levels of caspase-3 and cleaved caspase-3 expression in HBECs treated with different concentrations of avasimibe. **(D)** The intracellular CE ratio in HBECs. Data presented are means ± SD. n ≥ 3, *p < 0.05, **p < 0.001 by one-way ANOVA.

Avasimibe affects reduced intracellular cholesterol ester (CE) by specifically repressing ACAT1. We first investigated avasimibe’s efficacy in improving airway epithelial barrier disruption by analyzing the underlying mechanism of action *in vitro*. Summarily, primary mouse airway epithelial cells were successfully harvested as described (Supplementary Figure S2A). Limited by the count and the proliferation rate of primary basal cells, we could not utilize primary basal cells alone for further experiments, and we also found that the human bronchial epithelial cell lines (HBE-135°) mainly expressed the basal cell marker gene (Supplementary Figure S3A). Therefore, we used both the human bronchial epithelial cell line and primary basal cells for exploration of the mechanism of avasimibe during its action on improving barrier disruption. Results showed that cell viability declined with increasing avasimibe concentrations, while 0.5 µM of the drug had no effects on HBECs viability (Figure 3A). Primary basal cells treated with 0.5 µM avasimibe exhibited significantly improved HDM-induced proliferation, which was consistent with *in vivo* findings (Supplementary Figure S2B). On the other hand, treatment with 1 µM avasimibe resulted in significantly elevated apoptosis of HBECs (Figure 3B) and upregulation of cleaved caspase-3 (Figure 3C). However, avasimibe treatment did not lower the CE ratio intracellular in proper concentration (0.5 µM) (Figure 3D). Serum from mice in the HDM-induced group exhibited a significantly higher CE ratio than the control group, while those in the Ava-treated groups (20 and 30 mg/kg) had a significantly lower serum CE ratio than those in the HDM-induced group. However, we found no statistically significant differences in neither CE ratios of lung tissues (Figure 1F) nor total cholesterol across groups (Supplementary Figure S1B). Overall, these results demonstrated that certain concentrations of avasimibe do not significantly improve airway epithelial barrier disruption through the regulation of cholesterol metabolism.

### Upregulation of the KRT5 Gene Is Correlated With That of CTNNB1 in Severe Asthma

**FIGURE 4:**
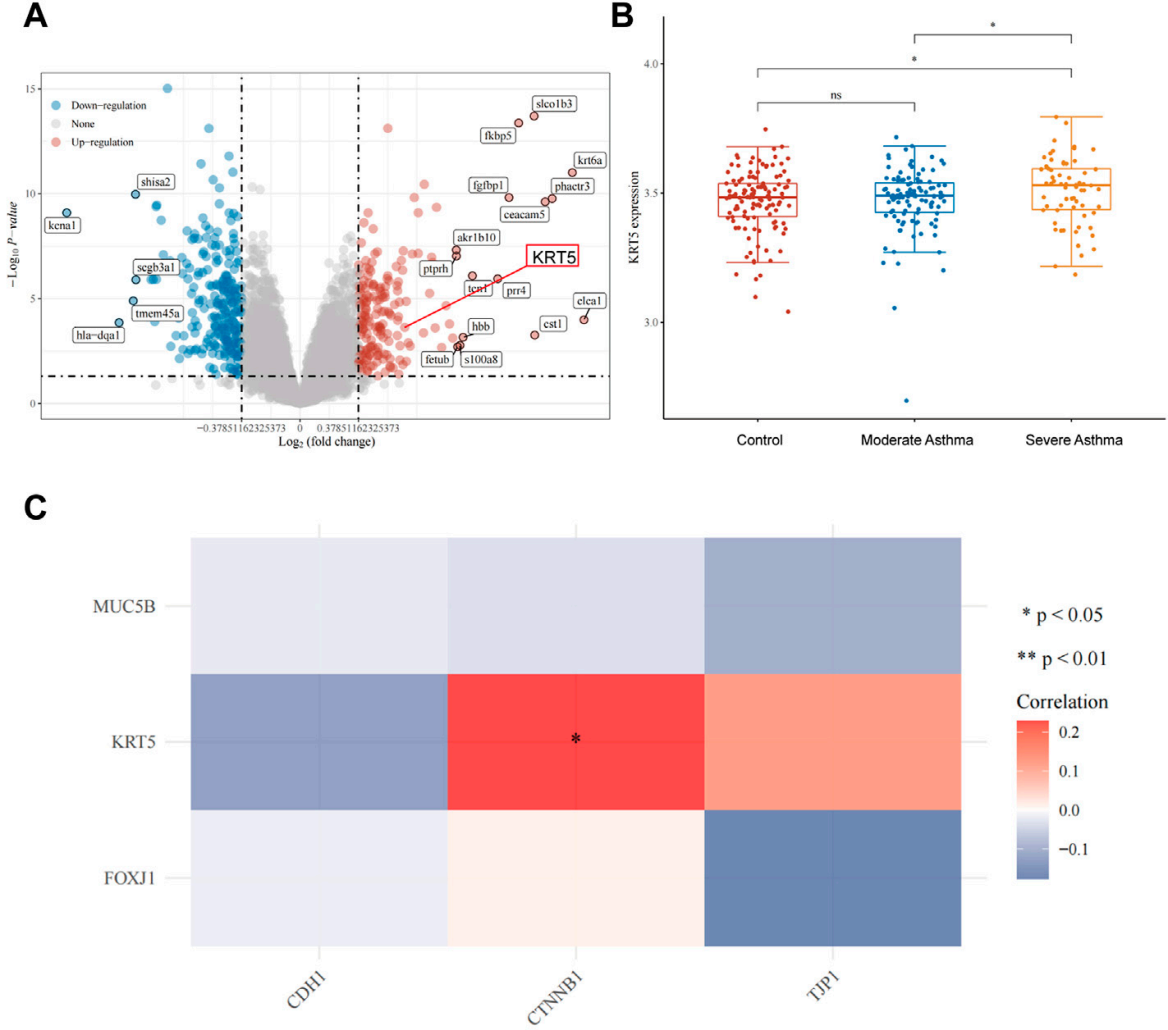
Upregulation of the KRT5 gene is correlated with β-catenin in severe asthma. **(A)** Volcano plots constructed using fold-change values (FC = 1.3) and adjusted P (*p* < 0.05). Red and blue dots represent significantly upregulated and downregulated mRNAs, respectively. **(B)** Distribution of KRT5 expression in the airway epithelium. The horizontal axis represents control, moderate asthma and severe asthma groups of samples, while the vertical axis denotes distribution of KRT5 expression. **(C)** Heat map showing the correlation between differentially expressed genes (MUC5B for goblet cell, KRT5 for basal cell, and FOXJ1 for ciliated cell) and epithelial barrier genes (CDH1 and CTNNB1 for adherens junctions, TJP1 for tight junctions). Horizontal and vertical axes represent differentially expressed genes, whereas different colors denote correlation coefficients. Red and blue representing positive and negative correlation, respectively. A darker color implies a stronger correlation. Asterisks represent levels of significance (*p < 0.05).

To further understand the association between avasimibe’s pharmacological effects and its role in improving epithelial barrier disruption, we analyzed three sequence datasets, namely, GSE43696, GSE64913, and GSE65584, using the Gene Expression Omnibus (GEO) database (https://www.ncbi.nlm.nih.gov/geo/). Volcano plots revealed a total of 8,194 differential expressed genes, of which 183 and 243 were upregulated (red dots) and downregulated (blue dots), respectively, in the airway epithelium of severe asthma group relative to the control group. As expected, the KRT5 gene was one of the upregulated genes (Figure 4A, red frame). Further analysis of KRT5 expression, across the three groups, showed that this gene was significantly upregulated in severe asthma compared with control and moderate asthma cases (Figure 4B). Overall, these data suggested that airway epithelial basal cells proliferated in severe asthmatic patients and were consistent with the earlier findings. Next, we found that KRT5 overexpression was strongly correlated with the expression of CTNNB1 (β-catenin) gene in severe asthmatic (Figure 4C). A review of previous literature revealed that the Wnt/β-catenin signaling pathway mediates proliferation of cells and growth of tissues, and the KRT5 is one of the most important markers of the airway epithelial basal cells, so the KRT5 upregulation in tracheas represented that airway epithelial basal cells were increased (Aman et al., 2011; Nusse and Clevers, 2017; Polverino et al., 2018). Moreover, recent research studies indicated that avasimibe inactivated the Wnt/β-catenin signaling pathway to suppress proliferation of cells by lowering Wnt secretion (Lee et al., 2018; Li et al., 2018). Therefore, we postulated that avasimibe exerts the same effects that suppress airway epithelial basal cell proliferation by blocking the Wnt/β-catenin signaling pathway in HDM-induced allergic inflammation.

### Avasimibe Suppresses Proliferation of Basal Cells and Improves β-Catenin Redistribution Independent on Targeting ACAT1

**FIGURE 5:**
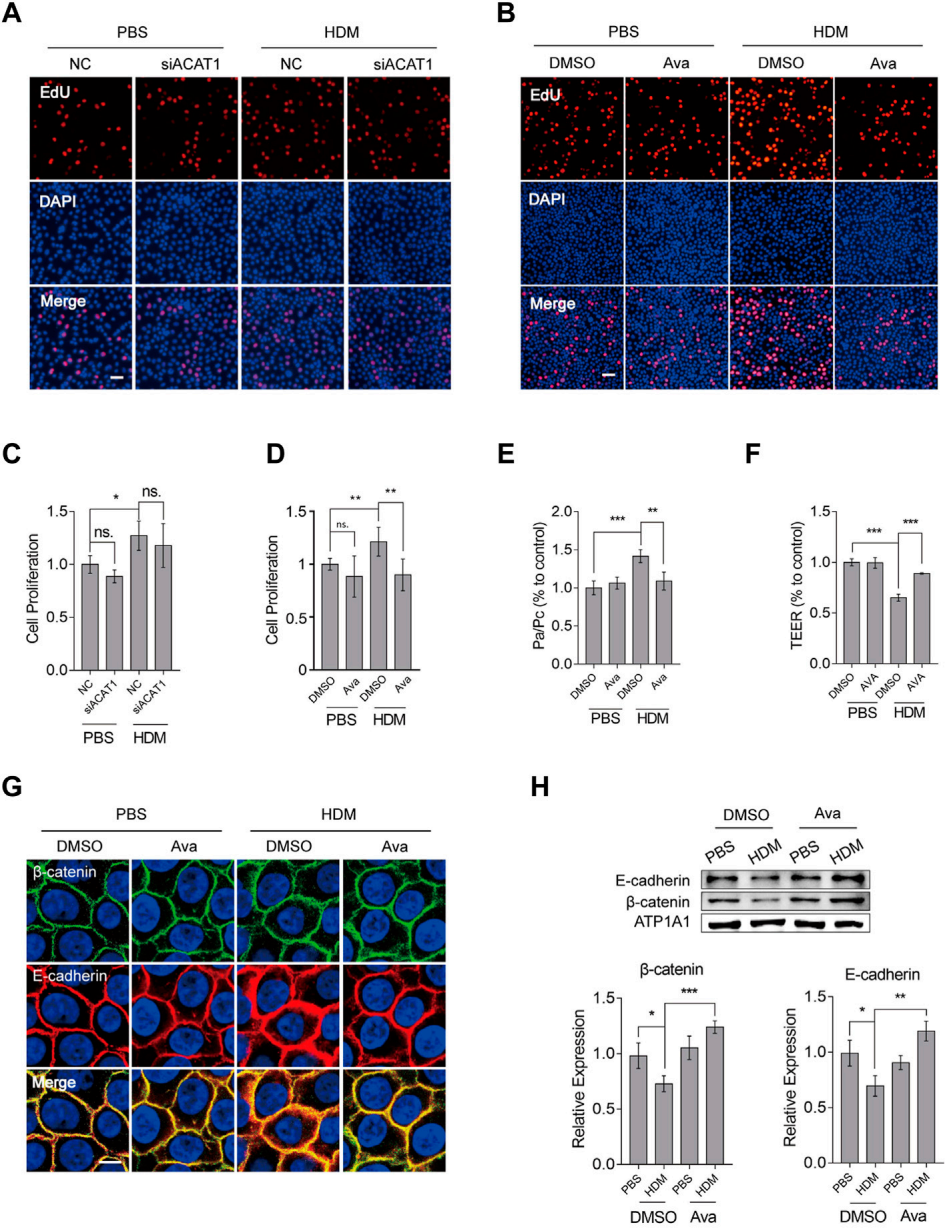
Avasimibe suppresses proliferation of HBECs and redistribution of β-catenin and E-cadherin. **(A)** Proliferation of HBECs based on EdU assay after silencing ACAT1. Scale bar = 100 µm. **(B)** Proliferation of HBECs based on the EdU assay after treatment with avasimibe (0.5 µM). Scale bar = 100 µm. **(C)** Proliferation of HBECs based on a CCK8 assay after silencing ACAT1. **(D)** Proliferation of HBECs based on the CCK8 assay after treatment with avasimibe (0.5 µM). **(E)** Assessment of epithelial barrier integrity using detection by fluorescein isothiocyanate (FITC) labeled dextrans (FITC-dextran) permeability. **(F)** Epithelial barrier integrity assessment using measurement of transepithelial electrical resistance (TEER). **(G)** Immunohistochemical staining showing distribution of β-catenin and E-cadherin in HBECs treated with avasimibe (0.5 µM). Scale bar = 10 µm **(H)** Expression profiles of β-catenin and E-cadherin proteins in membrane protein extracts in HBECs treated with avasimibe (0.5 µM). Data presented are means ± SD. n ≥ 3, *p < 0.05, **p < 0.001 by one-way ANOVA.

To validate our conjecture, we silenced ACAT1 and assessed the corresponding effects on HBEC proliferation using the EdU (Figure 5A) and CCK8 (Figure 5C) assays. Results from both experiments showed that targeting ACAT1 could not suppress HBEC proliferation. On the other hand, administration of avasimibe (0.5 µM) significantly suppressed proliferation on HBECs (Figure 5B, D) and primary basal cells (Supplementary Figure S2C). Notably, treatment with 0.5 µM avasimibe resulted in a similar pattern of KRT5 protein expression (Supplementary Figure S3B, C). These data showed avasimibe suppressed proliferation of HDM-induced basal cells but did not target ACAT1. To further investigate avasimibe’s effects on epithelial cells, we performed transepithelial electrical resistance (TEER) and FITC-Dx flux analyses to assess the function of epithelial barrier. Results revealed the significant improvement of HDM-induced epithelial barrier disruption, after treatment with 0.5 µM avasimibe (Figure 5E, F). Since our research group had previously found that HDMs induced β-catenin and E-cadherin redistribution, but not expression levels in the whole protein, we performed immunofluorescence and Western blot analyses to explore the distribution of both factors. Results showed that avasimibe alleviated HDM-induced redistribution of β-catenin and E-cadherin (Figure 5G) but had no effect on their expression levels based on results from whole protein expression (Supplementary Figure S3D). However, the drug increased the β-catenin expression level in the cytomembrane (Figure 5H). These data demonstrated that HDMs disrupted the epithelial barrier by inducing proliferation of basal cells and redistribution of adherens junction proteins, thereby avasimibe improving both of them.

### Avasimibe Lowered Proliferation of Basal Cells by Suppressing the Wnt/β-Catenin Signaling Pathway

**FIGURE 6:**
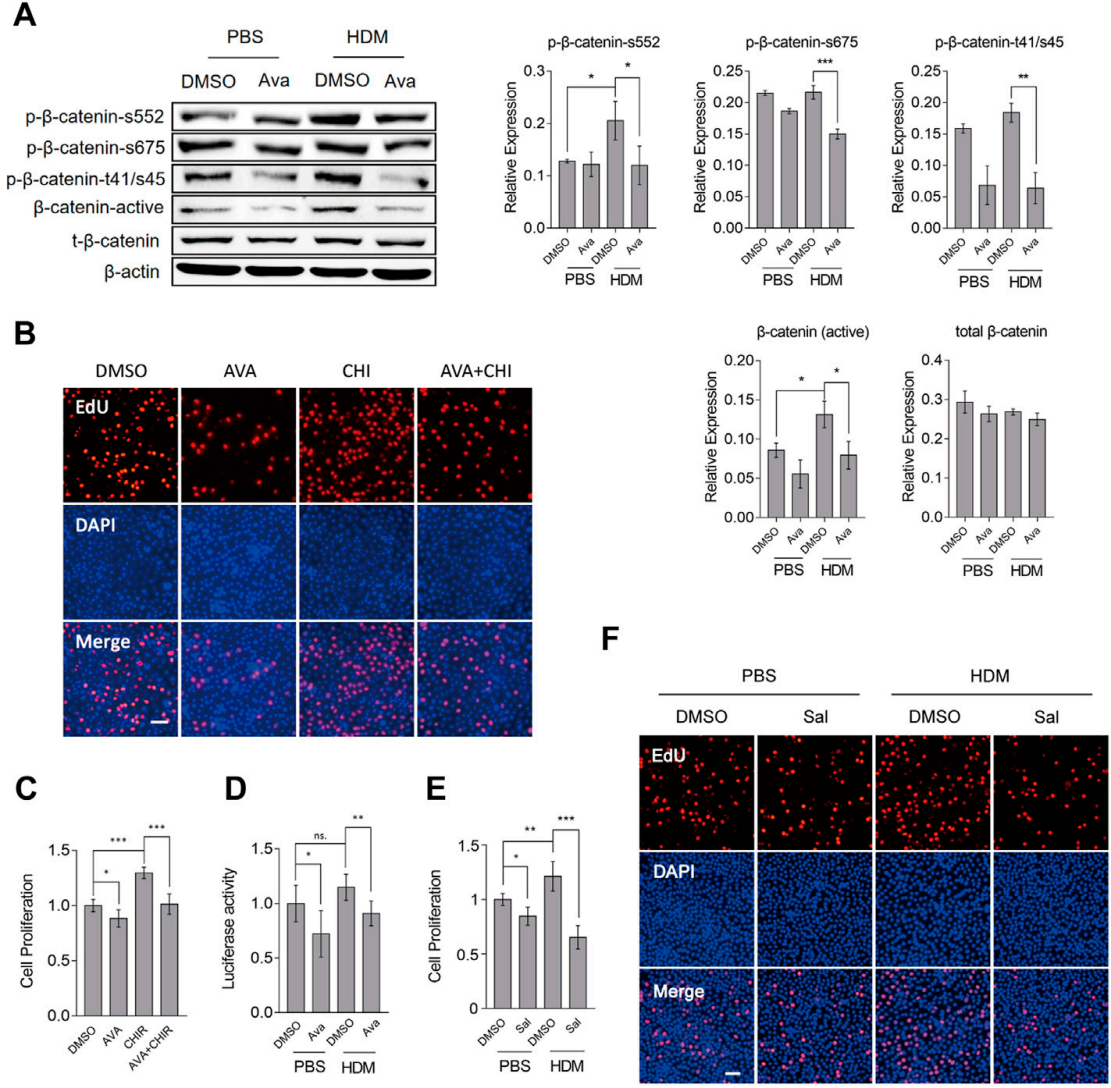
Avasimibe suppresses proliferation of HBECs by blocking the Wnt/β-catenin signaling pathway*.* **(A)** Western blots showing phosphorylation of β-catenin at Ser552, Ser675, and Thr41/Ser45, and active-β-catenin (non-phospho). **(B)** Proliferation of HBECs based on the EdU assay after treatment with avasimibe (0.5 µM) and/or CHIR99021 (5 nM, MCE, China), a potent activator of the Wnt/β-catenin signaling pathway. Scale bar = 100 µm **(C)** Proliferation of HBECs based on EdU assay after treatment with avasimibe (0.5 µM) and/or CHIR99021 (5 nM) **(D)** Assessment of Wnt/β-catenin signaling pathway activity. 1 μg/ml TOPFlash and 0.5 μg/ml active renilla luciferase mixed was employed as a reporter assay, and signals quantified as TOPFlash/Renilla ratio **(E)** Proliferation of HBECs based on EdU assay after treatment with salinomycin (0.5 µM, Selleck, United States), a potent inhibitor of Wnt/β-catenin signaling pathway. **(F)** Proliferation of HBECs based on EdU assay after treatment with salinomycin (0.5 µM). Scale bar = 100 µm. Data presented are means ± SD. n ≥ 3, *p < 0.05, **p < 0.001 by one-way ANOVA.

To ascertain whether avasimibe exerts its protective effect on the epithelial barrier by targeting the Wnt/β-catenin signaling pathway, we detected β-catenin-related phosphorylation sites. Previous studies have shown that these phosphorylation sites contribute to β-catenin localization at the cytomembrane, stabilization at the cytoplasm and increase cell proliferation (Ponce et al., 2011; Groulx et al., 2014; Zhu et al., 2019; Behrouj et al., 2021). Interestingly, avasimibe repressed phospho-β-catenin at Ser552, Ser675, and Thr41/Ser45 and also reduced active β-catenin levels, which could repress the Wnt/β-catenin activity (Figure 6A). To further confirm that avasimibe inactivated the Wnt/β-catenin activity, we used TOPFlash to detect the resultant effect on inactivation of the Wnt/β-catenin signaling pathway. Results showed that treatment with avasimibe (0.5 µM) significantly suppressed Wnt/β-catenin activation (Figure 6D). Next, we investigated the effect of CHIR99021 (CHIR), a potent Wnt/β-catenin agonist (Buikema et al., 2020), and found that treatment with avasimibe (0.5 µM) markedly decreased CHIR99021-induced cells proliferation, as evidenced by results of Edu and CCK8 assays, in both HBECs (Figure 6B, C) and primary basal cells (Supplementary Figure S2D). Furthermore, treatment of HBECs with salinomycin (Sal), a Wnt/β-catenin antagonist (Miyazaki et al., 1974), generated results that corroborated those from avasimibe administration (Figure 6E, F). Overall, these findings suggested that avasimibe impaired proliferation of basal cells by targeting β-catenin to inactivate the Wnt/β-catenin signaling pathway. Finally, we analyzed whether avasimibe reduced mucus secretion directly through avasimibe administration impaired differentiation of epithelial basal cells to goblet cells. To this end, we utilized A549, a lung epithelial adenocarcinoma cell line that can secrete mucus, to test our conjecture. As expected, results showed that avasimibe (0.5 µM) significantly suppressed HDM-induced mucus secretion (Supplementary Figure S3E). Taken together, these findings indicated that avasimibe alleviates the disruption of HDM-induced airway epithelial barriers by impairing the proliferation of basal cells and suppressing redistribution of adherens junction proteins, and reducing mucus secretion.

## Discussion

Repetitive chronic inflammation destroys the integrity of the airway epithelial barrier, thereby allowing environmental toxicants to reach the subepithelial tissue and further impairing airway tissue and exacerbating asthmatic symptoms (Jude et al., 2019). Our research has first focused on the association between cholesterol metabolism homeostasis and airway epithelial barrier disruption in asthma, since the former plays a critical role in inflammatory disorder. During the course of our study, we administered three different concentrations of avasimibe (10, 20 and 30 mg/kg) to ascertain the optimal concentration that effectively alleviates allergic asthma symptoms. Interestingly, 20 mg/kg avasimibe had the best effects, significantly alleviating HDM-induced airway inflammation (Figure 1E, G) and improving HDM-induced airway resistance through reducing airway smooth cells hyperplasia (Supplementary Figure S5). This regimen also improved disruption of the airway epithelial barrier *in vivo*, including reduced basal and goblet cells proliferation, mucus hypersecretion, and suppressed redistribution of adherens junction proteins in epithelial cells, although this effect in the lung tissue was independent of its main pharmacological function, which is regulating cholesterol metabolism.

Except for the HDM-induced asthmatic mouse model *in vivo*, we successfully harvested primary airway epithelial basal cells for *in vitro* investigations to further validate our findings. We used both human bronchial epithelial cell line and primary airway epithelial basal cells for the following explorations, and results showed that avasimibe exerted the effects of impairing proliferation of basal cells and suppressing the distribution of β-catenin, and these effects seemed consistent with the results *in vivo*. Those phenomena caught our eye, considering results *in vivo* and *in vitro*; we hypothesized that avasimibe exhibited those effects through other mechanisms but not its main pharmacological function of targeting ACAT1 to repress cholesterol ester accumulation in cells.

Avasimibe shows an excellent safety profile in treating atherosclerosis by reducing the accumulation of cholesterol ester in foam cells (Pal et al., 2013). However, the lung is an organ with less cholesterol, and epithelial cells have less cholesterol than foam cells; those may be the main reasons why avasimibe exerted no effects of cholesterol ester reduction in the lung or epithelial cells. The airway epithelial basal cells regulate functioning of stem cells to maintain airway epithelial integrity, owing to their capacity to proliferate and differentiate into various epithelial cells. However, the repair of airway epithelia is prone to metaplasia in disease conditions, which is one of the reasons for airway remodeling and mucus hypersecretion. Interestingly, avasimibe also exerted its effects in airway submucosa in our research, but this phenomenon has rarely been reported in current studies; the hyperplasia of smooth cells is one of the most important characteristics in airway remodeling in asthma. Thereby, the multiple effects of avasimibe in HDM-induced asthma surprised us indeed; in the present study, we have focused on the proliferation of airway epithelial basal cells, and the effects of avasimibe in smooth cells maybe a direction for our future investigation.

Our group has engaged the mechanism underlying impairing and repairing of the airway epithelial barrier in HDM-induced allergic asthma. Our previous studies have demonstrated that HDMs induced adherens junction proteins (β-catenin and E-cadherin) redistribution resulted in the disruption of airway epithelial barrier (Dong et al., 2016; Dong et al., 2017; Zhang et al., 2017; Ye et al., 2019). In the present study, we focused on the airway basal cells which have the proliferation and differentiation abilities as stem cells to maintain the integrity of airway epithelial barrier. Although wound tissues repaired after disruption increase cell proliferation and differentiation to compensate the desquamated tissues, the epithelium is prone to metaplasia, which explains why goblet cell hyperplasia and metaplasia lining the airway epithelium cause mucus hypersecretion (Freishtat et al., 2011). Accumulating evidence has shown that the basal cells proliferate and differentiate to become other epithelial cells and maintain the integrity of the airway epithelium after disruption. However, the dysregulation of epithelial basal cells is responsible for the abnormal remodeling process in severe asthma, characterized by hyperplasia of basal and goblet cells, and squamous cell metaplasia (Maouche et al., 2009). To date, the underlying mechanism of the aberrant proliferation of airway epithelial basal cells in asthma remains unknown.

To elucidate the actual mechanism through which avasimibe suppresses proliferation and metaplasia of basal cells, we analyzed sequence datasets from the GEO database and then correlated expression patterns of KRT5 and CTNNB1 genes (coding for β-catenin protein). These results indicated that airway epithelial basal cell proliferation is correlated with β-catenin. There is also accumulating evidence that has shown that the Wnt/β-catenin signaling pathway mediates cell proliferation and tissue growth (Nusse and Clevers, 2017), while avasimibe can repress this pathway (Li et al., 2018). To exclude the interference of others factors, the Wnt/β-catenin signaling agonist and inhibitor, CHIR99021 and salinomycin, were used to validate that avasimibe inhibited the Wnt/β-catenin signaling pathway in the present study, respectively (Figure 6B, C, E, F, Supplementary Figure S2D). Therefore, we postulated that avasimibe could improve the disruption of the airway epithelial barrier by inactivating the Wnt/β-catenin signaling pathway.

Experimental results indicated that avasimibe treatment localized β-catenin to the membrane, dephosphorylated and inactivated β-catenin to exert its function on the Wnt/β-catenin signaling pathway. Moreover, avasimibe suppressed proliferation of both HBECs and primary basal cells by inactivating the Wnt/β-catenin signaling pathway. In addition, avasimibe treatment significantly reduced the proliferation of epithelial basal cells and suppressed redistribution of adherens junction proteins, thereby alleviating the disruption of the airway epithelial barrier, both *in vivo* and *in vitro*. Notably, in the present study, we could not unravel the deeper level mechanism underlying avasimibe’s effect on how to regulate the Wnt/β-catenin signaling pathway; thus, further explorations are needed to ascertain whether this occurs directly or through some other complex mechanism.

Treatments for allergic asthma is intractable possibly because any changes in asthmatic symptoms are likely to impact prevalence and treatment outcomes. Currently, inhaled corticosteroids are the main therapy for asthmatic patients and have shown outcomes (Sharma et al., 2015). However, at present, only a handful of etiological treatments have been developed, with most of these merely focusing on relieving symptoms and delaying asthma progression (Dhariwal et al., 2017).

In conclusion, our findings demonstrated that avasimibe is a potent medicine for alleviating disruption of the airway epithelial barrier, with an excellent safety profile. Therefore, it is a promising target for clinical therapies for the treatment of allergic asthma.

## Data Availability

The datasets presented in this study can be found in online repositories. The names of the repository/repositories and accession number(s) can be found in the article/Supplementary Material.
