# Supplementary material for: Avasimibe Alleviates Disruption of the Airway Epithelial Barrier by Suppressing the Wnt/β-Catenin Signaling Pathway
Source: Front Pharmacol. 2022 Feb 11;13:795934. doi: 10.3389/fphar.2022.795934 (PMC8874122; doi:10.3389/fphar.2022.795934)
Supplement: Supplementary file 1 [file DataSheet1.PDF]

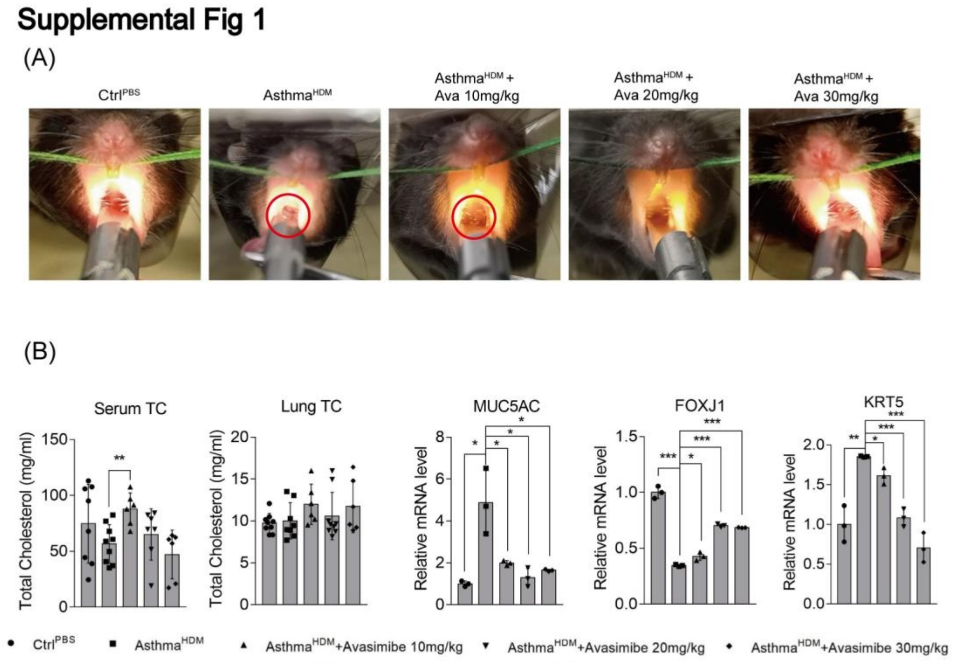

Supplementary Figure 1. Effects of different Avasimibe concentrations in vivo.

(A)Presence of mucus secretion through the oropharynx of anesthetized mice. (B)Concentrations of total cholesterol (TC) in serum and lung tissues treated with different concentrations of Avasimibe. (C)qPCR results showing mRNA levels of MUC5AC (goblet cell), FOXJ1 (ciliated cell) and KRT5 (basal cell) in lung treated with different concentrations of Avasimibe. Different shape dots represent different groups. Data presented are means±SD. n≥3, *P＜0.05, **P＜0.001 by one-way ANOVA.t.

Supplemental Fig 2

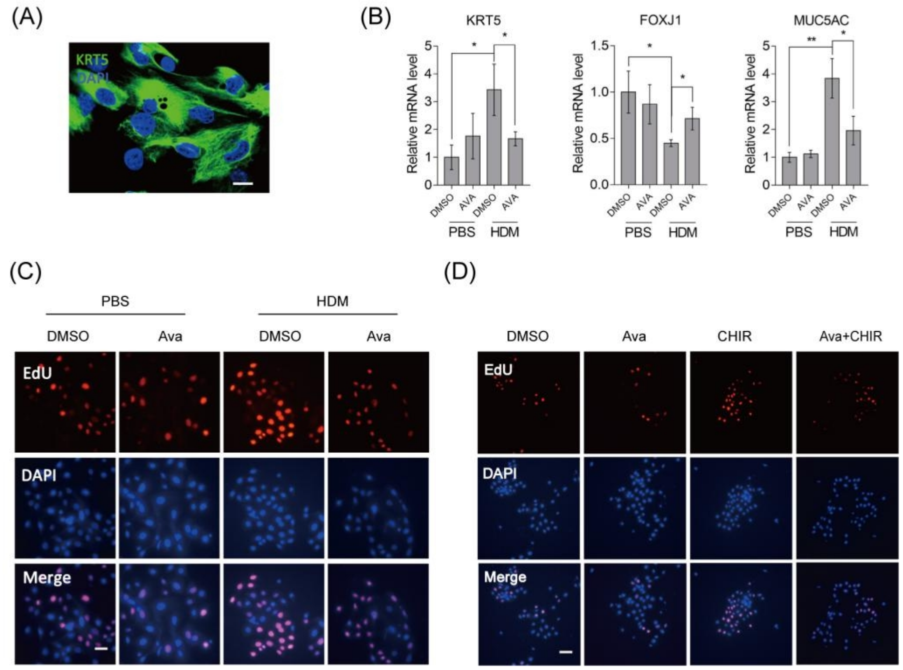

Supplementary Figure 2. Avasimibe suppresses barrier disruption through reduced epithelial basal cells proliferation.

(A)Immunofluorescence staining was used to detect airway epithelial basal cells. (B)qPCR results showing mRNA levels of MUC5AC (goblet cell), FOXJ1 (ciliated cell) and KRT5 (basal cell) in primary basal cells. (C)The EdU assay was used to assess the proliferation of primary cells administration with Avasimibe. Scale bar = 100 µm. (D)The EdU assay was used to assess the proliferation of primary cells administration with Avasimibe and/or CHIR99021. Scale bar = 200 µm. Data presented are means±SD. n≥3, *P＜0.05, **P＜0.001 by one-way ANOVA.

Supplemental Fig 3

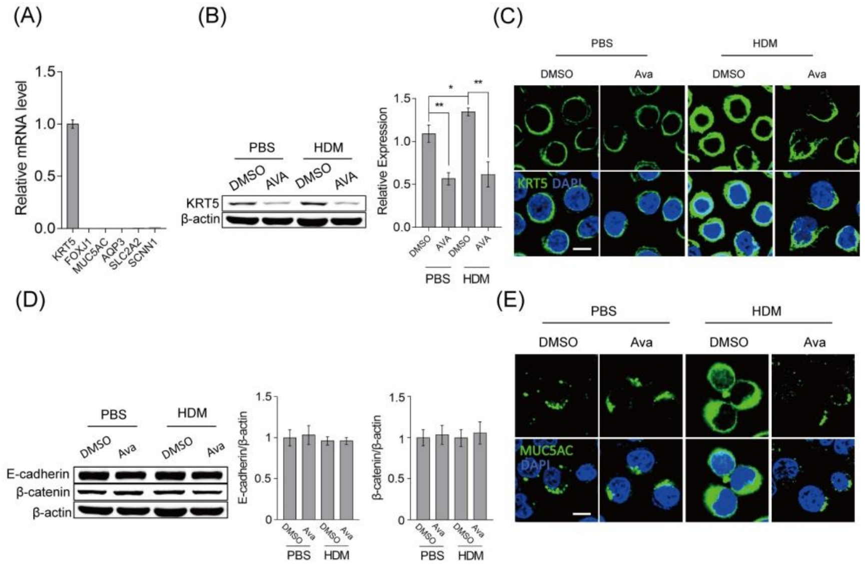

Supplementary Figure 3. Effects of Avasimibe in different cell lines.

(A)qPCR results showing basal cell features in HBE-135o. KRT5 for basal marker; FOXJ1 for cilia marker; MUC5AC for mucus marker; AQP3 for water channel marker; SLC12A2 for transporter channel marker; SCNN1 for ion channel marker. (B)Profiles of KRT5 expression in whole protein extract. (C)Immunofluorescence staining with KRT5 in HBECs administration with Avasimibe. Scale bar = 10 μm. (D)Profiles of β-catenin and E-cadherin expression in whole protein extract. (E)Immunofluorescence staining with MUC5AC in A549 administration with Ava. Scale bar = 10 μm. Data presented are means±SD. n≥3, *P＜0.05, **P＜0.001 by one-way ANOVA.

Supplemental Fig 4

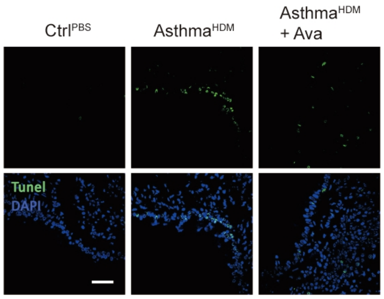

Supplementary Figure 4. Apoptosis of resident lung cells with different treatments.

Detection of TUNEL-positive cells, represented apoptosis cells, in lung tissues by TUNEL staining. Green for TUNEL, blue for DAPI. Scale bar = 200 μm

Supplemental Fig 5

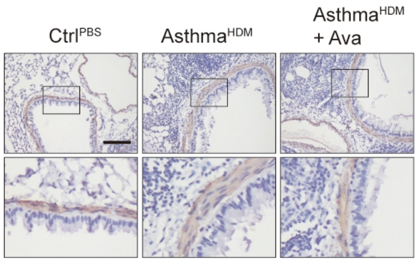

Supplementary Figure 5. Avasimibe reduced hyperplasia of smooth cells in airway submucosa.

Immunohistochemistry was used to detect α-SMA in airway submucosa. Scale bar = 200 μm
